# Phenotyping asthma using an unsupervised prediction model based on blood granulocyte responsiveness

**DOI:** 10.1186/2045-7022-5-S2-O2

**Published:** 2015-03-23

**Authors:** Bart Hilvering, Susanne Vijverberg, Jeroen Jansen, Leo Houben, Rene Schweizer, Jan-Willem Lammers, Leo Koenderman

**Affiliations:** 1 University Medical Centre Utrecht, Respiratory Medicine, Lab. of Trans. Immunology, Utrecht, Netherlands; 2 Utrecht Institute for Pharmaceutical Sciences (UIPS), Faculty of Science, Division of Pharmacoepidemiology & Clinical Pharma, Utrecht, Netherlands; 3 Radboud University, IMM, Nijmegen, Netherlands; 4 University Medical Centre Utrecht, Respiratory Medicine, Utrecht, Netherlands

## Background

The clinical identification of inflammatory asthma phenotypes currently requires sputum analysis. Sputum analysis has proven its value in diagnosis and disease monitoring. However, technical limitations hamper its implementation in daily clinical practice. There is a strong need for fast, non-invasive and accurate diagnostic methods to phenotype and monitor asthma.

## Objective

To assess whether Nonlinear Principal Component Analysis (NLPCA) of peripheral blood characteristics and clinical parameters can identify inflammatory phenotypes in asthma without the need for sputum analysis.

## Method

Clinical parameters, activation of blood granulocytes and sputum phenotypes were assessed in 115 adult asthma patients (NCT01611012). Blood granulocytes were stained with antibodies against the active FcγRII receptor (CD32, clones A17/A27) and α-chain of MAC-1 (CD11b) in the presence or absence of fMLF, and analysed by flow cytometry. NLPCA was used to reduce dimensions in a combined cellular and clinical dataset, followed by discriminant analysis to generate a prediction model. Phenotypes identified by the model were cross-validated by inflammatory sputum phenotypes based on sputum induction (reference test).

## Results

Peripheral blood eosinophil count, FeNO (Fraction of Exhaled Nitric Oxide), ACQ (Asthma Control Questionnaire), medication use, nasal polyposis, aspirin sensitivity and neutrophil/eosinophil responsiveness upon stimulation, are important parameters to differentiate between eosinophilic and non-eosinophilic phenotypes. The combinations of these parameters lead to an accurate prediction model for eosinophilic asthma with 90.5% sensitivity and 91.5% specificity.

## Conclusion

The proposed prediction model identifies eosinophilic asthma by using peripheral blood analysis, FeNO and routine clinical data, resulting in a reliable non-invasive test that is adequate for daily clinical practice.

